# Structure, Function and Protein Engineering of Cereal-Type Inhibitors Acting on Amylolytic Enzymes

**DOI:** 10.3389/fmolb.2022.868568

**Published:** 2022-03-25

**Authors:** Marie Sofie Møller, Birte Svensson

**Affiliations:** ^1^ Applied Molecular Enzyme Chemistry, Department of Biotechnology and Biomedicine, Technical University of Denmark, Kgs. Lyngby, Denmark; ^2^ Enzyme and Protein Chemistry, Department of Biotechnology and Biomedicine, Technical University of Denmark, Kgs. Lyngby, Denmark

**Keywords:** CM-proteins, proteinaceous inhibitor, enzyme complexes, binding constant, x-ray crystallography, insect pests, limit dextrinase, food and nutrition

## Abstract

Numerous plants, including cereals, contain seed proteins able to inhibit amylolytic enzymes. Some of these inhibitors, the CM-proteins (soluble in chloroform:methanol mixtures)—also referred to as cereal-type inhibitors (CTIs)—are the topic of this review. CM-proteins were first reported 75 years ago. They are small sulfur-rich proteins of the prolamine superfamily embracing bifunctional *α*-amylase/trypsin inhibitors (ATIs), *α*-amylase inhibitors (AIs), limit dextrinase inhibitors (LDIs), and serine protease inhibitors. Phylogenetically CM-proteins are predicted across poaceae genomes and many isoforms are identified in seed proteomes. Their allergenicity and hence adverse effect on humans were recognized early on, as were their roles in plant defense. Generally, CTIs target exogenous digestive enzymes from insects and mammals. Notably, by contrast LDI regulates activity of the endogenous starch debranching enzyme, limit dextrinase, during cereal seed germination. CM-proteins are four-helix bundle proteins and form enzyme complexes adopting extraordinarily versatile binding modes involving the N-terminal and different loop regions. A number of these inhibitors have been characterized in detail and here focus will be on target enzyme specificity, molecular recognition, forces and mechanisms of binding as well as on three-dimensional structures of CM-protein–enzyme complexes. Lastly, prospects for CM-protein exploitation, rational engineering and biotechnological applications will be discussed.

## 1 Introduction

Proteinaceous *α*-amylase inhibitors belonging to different protein families; knottins, defensins, Kunitz-type inhibitors, CM-proteins, legume lectins, *γ*-thionins, lipid-transfer proteins, xylanase-*α*-amylase inhibitory proteins, and thaumatin-like inhibitors are mostly found in plants, although some occur in mollusks and microorganisms. This kind of *α*-amylase inhibitors were recognized long ago. They have been collectively covered in several reviews as well as in recent publications on specific inhibitors presenting rather different levels of structural and mechanistic insights (Kneen and Sandstedt, 1946; Garcia-Olmedo et al., 1987; Blanco et al., 1991; Carbonero and García-Olmedo, 1999; Svensson et al., 2004; Juge and Svensson, 2006; de Oliveira Carvalho and Gomes, 2009; Rehm et al., 2009; dos Santos et al., 2010; Kumar et al., 2010; Wang et al., 2014; Gadge et al., 2015; Sun et al., 2015; da Silva et al., 2018; Panwar et al., 2018; Tysoe and Withers, 2018; Tsvetkov and Yarullina, 2019; Juhász et al., 2020; Rane et al., 2020; Aguieiras et al., 2021; Geisslitz et al., 2021). The biological role of the plant hydrolase inhibitors is primarily in defense against insect pests and pathogenic fungi, whereas they are rarely involved in regulation of the activity of endogenous plant enzymes. Hydrolase inhibitors of certain protein families can be bifunctional and act both on amylolytic enzymes of glycoside hydrolase family 13 (GH13) (Drula et al., 2022) and serine proteases, while other members of the same families only inhibit either amylolytic enzymes of GH13 or serine proteases (Barber et al., 1986; Carbonero and Garcia-Olmedo, 1999; di Maro et al., 2011). In several cases, the dual enzyme inhibition has been experimentally confirmed along with corresponding three-dimensional structures and models of enzyme–double-headed plant inhibitor complexes (Mundy et al., 1983; Maskos et al., 1996; Strobl et al., 1998; Vallée et al., 1998; Micheelsen et al., 2008; Grosse-Holz and van der Hoorn, 2016). The topic of the present review is the family of cereal-type inhibitors (CTIs), in particular inhibitors of amylolytic enzymes, which have been first described 75 years ago (Kneen and Sandstedt, 1946). CTIs are all found in cereals and other grass species and can amount to 2–4% of the seed protein content. These inhibitors belong to the prolamine superfamily of plant proteins and are called CM-proteins after their solubility in chloroform:methanol mixtures (Carbonero and García-Olmedo, 1999; Mills et al., 2004; Geisslitz et al., 2021). Some CM-proteins, referred to *a*-amylase/trypsin inhibitors—or ATIs for short—display bifunctionality and have two target enzymes. This protein family also contains monofunctional inhibitors against *α*-amylases from insects and mammals as well as the starch debranching enzyme limit dextrinase, which all belong to GH13, and similarly other members only inhibit serine proteases. Notably, CM-proteins also receive major attention due to their behavior as antinutrients and allergens harmful to human health including non-celiac wheat sensitivity (NCWS) and Bakers’ asthma (Wang et al., 2014; Reig-Otero et al., 2018) (for a review see Geisslitz et al., 2021). However, our focus will be on biochemical and structural properties of CM-protein inhibitors, i.e. their target enzyme specificity and inhibition kinetics, affinity and mechanism of enzyme binding as well as on three-dimensional structures of complexes with enzymes of family GH13. Some of these cases can provide a basis for using rational protein engineering to develop improved inhibitors for various potential applications.

## 2 General Characteristics, Occurrence and Phylogeny of CM-Proteins

**FIGURE 1:**
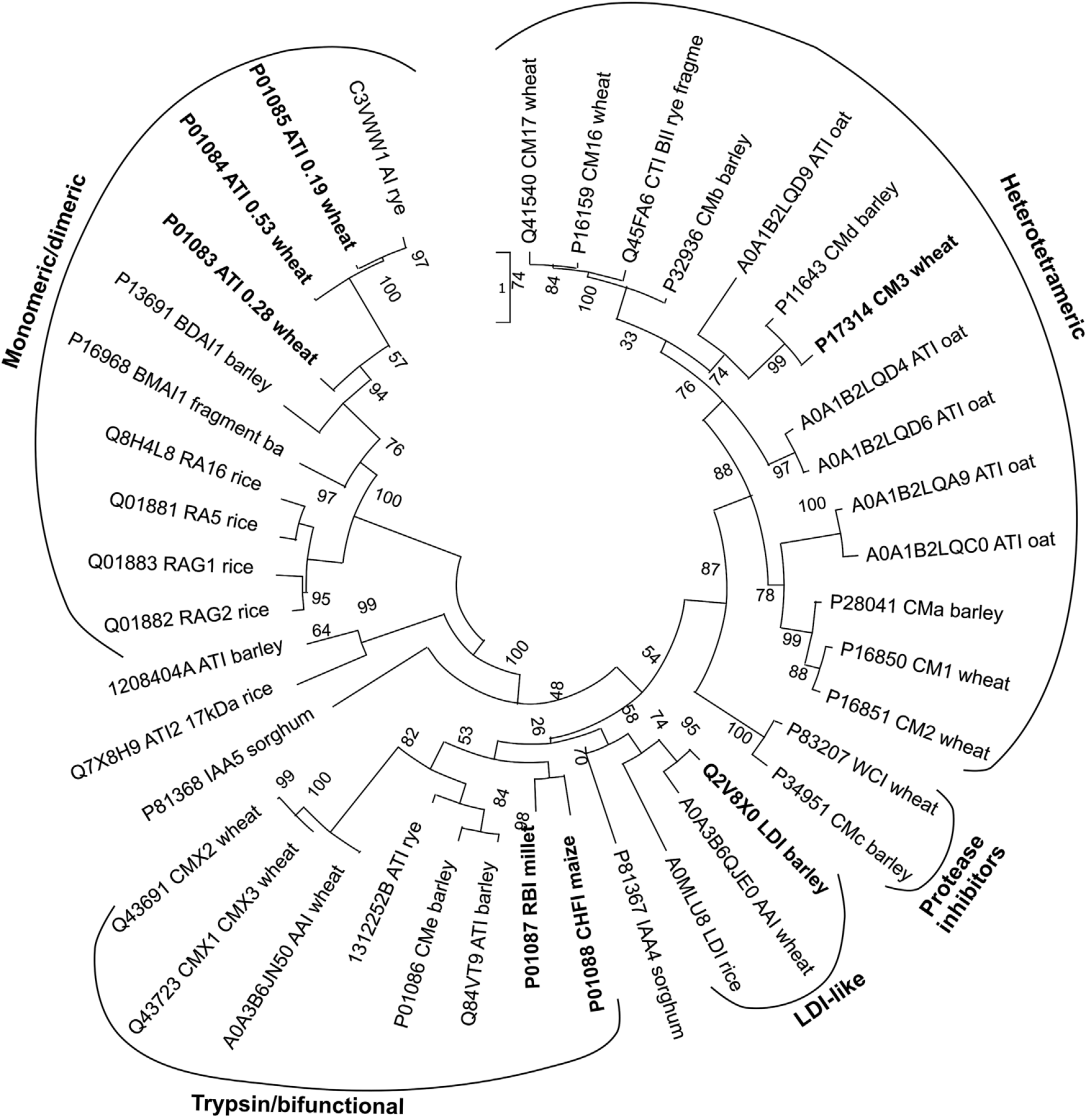
Phylogenetic analysis of characterized CTIs and homologues from other plants. The well-characterized proteins included in Table 1 are shown in bold. Names and origin of the proteins are indicated. The sequences were retrieved from UniProt database (1 February 2022). Software used: Promals3D (Pei et al., 2008) for structure-guided multiple alignment and MEGA 11 (Kumar et al., 2018) for Maximum likelihood for phylogeny analysis. Bootstrap values for 1,000 replicates are shown.

**FIGURE 2:**
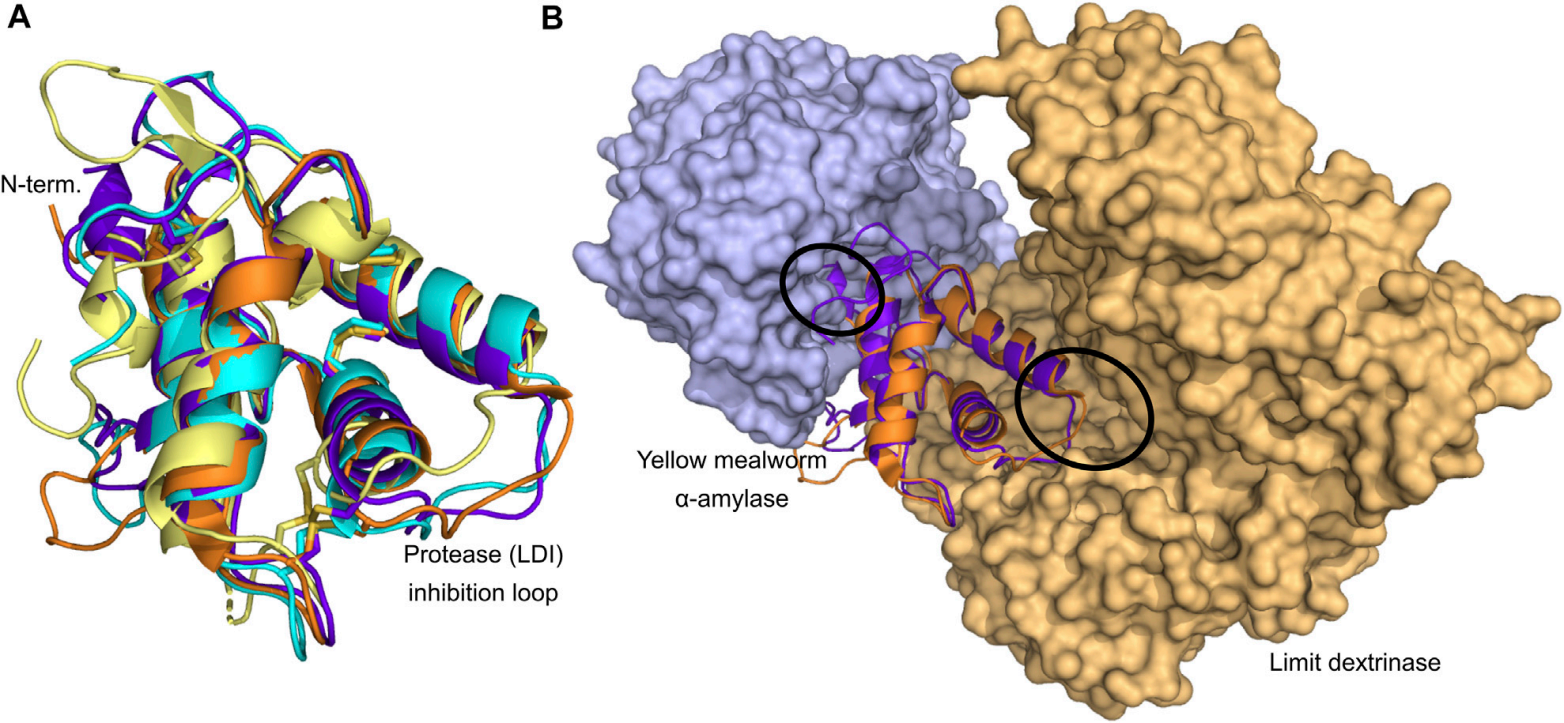
Structure determined CTIs. **(A)** Structural superposition of the four structure determined CTIs; LDI (orange; PDB entry 4CWV), RBI (purple; PDB entry 1TMQ), CHFI (cyan; PDB entry 1BEA), and 0.19 (yellow; PDB entry 1HSS). Disulfide bonds are shown as sticks. **(B)** Comparison of the inhibitor orientation of the two available CTI–enzyme complexes. LDI (orange; PDB entry 4CWV) and RBI (purple; PDB entry 1TMQ) are superposed revealing that opposite sides of the inhibitors are involved in the inhibition. The active sites of the enzymes are encircled. Structures were retrieved from the Protein Data Bank (PDB; www.rcsb.org).

The CM-proteins are small proteins of 12–16 kDa containing four to five well-conserved disulfide bonds and can be either monomeric or composed of two or four subunits (Carbonero and García-Olmedo, 1999). They are found in seeds of a wide range of cereal crops; wheat, barley, oats, rye, finger millet, barnyard millet, corn, rice, and sorghum (Garcia-Olmedo et al., 1987; Feng et al., 1991; Chen et al., 1992; Maskos et al., 1996; Carbonero and García-Olmedo, 1999; Altenbach et al., 2011; Wang et al., 2014; Gadge et al., 2015; Gazza et al., 2016; Panwar et al., 2018; Sagu et al., 2020). Many isoforms have been identified for example in barley and wheat (Østergaard et al., 2004; Guo et al., 2016; Bose et al., 2020; di Francesco et al., 2020; Geisslitz et al., 2020). In wheat the more prominent ones are 0.28 (monomeric), 0.19 and 0.53 (both homodimeric) (named based on electrophoretic mobility), CM1, CM2, CM3, CM16 and CM17 (all heterotetrameric) (for names and numbering see Rodriguez-Loperena et al., 1975; Carbonero and Garcia-Olmedo, 1999; Geisslitz et al., 2021). A time lag between ATI accumulation during wheat grain filling and detection of the biological activity suggested that assembly into dimers and tetramers determined the inhibitory potential (Call et al., 2021). Nineteen ATI isoforms from the wheat cultivar Butte 86 (Altenbach et al., 2011) and 33 proteoforms of ATIs across different bread wheat cultivars are reported (Bose et al., 2020; Geisslitz et al., 2021). These comprehensive analyses of wheat reflect the interest in CM-proteins due to the impact they may have on human health, albeit some address their role in plant defense as *α*-amylase or protease inhibitors, while the studies on barley also concerned protein mapping of cultivars for malting and beer brewing (Østergaard et al., 2004; Iimure et al., 2015; Guo et al., 2016; Perlikowski et al., 2016; Bose et al., 2019). Different proteoforms of CM-proteins in wheat and food products in light of possible implications in NCWS were determined by using advanced mass spectrometry (Bose et al., 2020; Geisslitz et al., 2020). Related to consumers’ interest in ancient cultivars, it has been noted that the CM-protein contents in old and modern Italian durum wheat genotypes, showed most isoforms to be shared, although a couple were only identified in an ancient cultivar (di Francesco et al., 2020). As mentioned above, the isoforms occur in different states of oligomerisation, the monomeric show high inhibitory activity on insect *α*-amylases, the homodimeric inhibitors react well with both insect and mammalian *α*-amylases, while the heterotetrameric inhibitors are highly active towards insect *a*-amylases (Table 1) (Carbonero and García-Olmedo, 1999; Franco et al., 2002). In addition to the CTIs inhibiting *α*-amylases, a small group of CTIs only act on serine proteases. WCI (wheat chymotrypsin inhibitor) is a strong inhibitor of bovine pancreatic chymotrypsin as well as of chymotryptic-like activities isolated from cotton bollworm and yellow mealworm (*Tenebrio molitor*), while no inhibition was detected against bovine pancreatic trypsin, or *α*-amylases from yellow mealworm (TMA) and human saliva (HSA) (di Maro et al., 2011). Barley CMc (equivalent to WCI) and CMe inhibited trypsin, but not TMA. Only CMa inhibited TMA among the CMa–e proteins from barley (Barber et al., 1986). Because a large number of different CM-proteins and posttranslationally modified forms thereof are present in seeds, it is difficult to purify any of the proteins to a highly homogenous state from natural sources for characterization of structure and function. Therefore, selected CM-proteins have been produced recombinantly in microbial hosts, *Escherichia coli* (CM2, CM3, CM16, 0.28, corn Hageman factor inhibitor, bifunctional *α*-amylase/trypsin inhibitor, and rye BIII) and *Pichia pastoris* (LDI, CM3, CM16, and 0.28) (García-Maroto et al., 1991; Strobl et al., 1995; Behnke et al., 1998; Kusaba-Nakayama et al., 2001; Dias et al., 2005; Jensen et al., 2011; Tundo et al., 2018) or in lentivirus transfected human embryonic kidney cells (CM3, the most prominent isoform in wheat) (Thiel et al., 2020). While recombinant CTIs were not applied in clinical testing (Geisslitz et al., 2021), evaluation of allergenicity has been performed in cellular assays (Tundo et al., 2018) and the effect on gut microbiota in *Drosophila melanogaster* (Thiel et al., 2020). Studies in rats and using caco-2 cells showed enhanced absorption rate for the abundant isoform CM3 as compared to CM16 and 0.28 from wheat (Kusaba-Nakayama et al., 2001). Phylogenetic analyses reveal that the monomeric and dimeric wheat CTIs are closely related (Figure 1), while the bifunctional CTIs are more related to the LDI-like CTIs than the other CTI groups (Behnke et al., 1998; Møller et al., 2015; Geisslitz et al., 2021). The sequence conservation of the CTIs is very low (Figure 1), but comparison of the four CTIs for which 3D structures have been determined shows that the structural conservation is very high (Figure 2A).

**TABLE 1:** Biochemically well-characterized cereal type inhibitors. The Protein Data Bank (PDB) entries are given for structure-determined proteins. Abbreviations of enzymes mentioned in the table: HPA, human pancreatic *α*-amylase; HSA, human salivary *α*-amylase; LD, barley limit dextrinase; PPA, porcine pancreatic *α*-amylase; TMA, yellow mealworm *α*-amylase.

Source	Protein Name	Identified target(s)	Confirmed Lack of Inhibition	PDB Entry	References
Barley (*Hordeum vulgare*)	Limit dextrinase inhibitor (LDI)	LD (*K*_D_ = 0.042 nM) Very limited inhibition of *Klebsiella pneumoniae* pullulanase and *Pseudomonas amyloderamosa* isoamylase	*Bacillus acidopullulyticus* pullulanase, malted barley *α*-amylase, TMA, PPA, trypsin	4CVW	(MacGregor et al., 1994, 2000; Stahl et al., 2007; Jensen et al., 2011; Møller et al., 2015, 2021)
Emmer (*Triticum dicoccon*)	Heterotetrameric (CM2, CM3x2, CM16) *α*-amylase inhibitor (ETI)	PPA (*K*_i_ = 1.82 nM), HSA (*K*_i_ = 3.25 nM), TMA	*B. subtilis* and barley *α*-amylases		Capocchi et al. (2013)
	E-WMAI (0.28)	TMA, HSA, and *α*-amylases from red flour beetle, rice weevil and Mediterranean flour moth			Capocchi et al. (2021)
Maize (*Zea mays*)	Corn Hageman factor/α-amylase inhibitor (CHFI)	TMA *α*-amylase from red flour beetle Hageman factor (Factor XIIa) (*K*_i_ = 1.0 nM), Factor XIa (*K*_i_ = 5.4 µM), bovine pancreatic trypsin (*K*_i_ = 2.1 nM), mammalian trypsins, trypsin from yellow mealworm	α-amylases from rice weevil	1BEA	(Mahoney et al., 1984; Chong and Reeck, 1987; Chen et al., 1992; Behnke et al., 1998; Korneeva et al., 2014)
Ragi/Indian finger millet (*Eleusine coracana*)	Ragi bifunctional *α*-amylase/trypsin inhibitor (RBI/RATI/RABI)	TMA, PPA (*K*_i_ = 11 nM, substrate dependent) Bovine trypsin (*K*_i_ = 1.2 nM)		1B1U 1BIP 1TMQ	(Strobl et al., 1995, 1998; Maskos et al., 1996; Gourinath et al., 2000; Alam et al., 2001)
Rye (*Secale cereale*)	BIII	PPA (low), HSA (low), and *α*-amylases from bean weevils and cotton boll weevil	Bovine pancreatic trypsin or boll weevil trypsin		(Iulek et al., 2000; Oliveira-Neto et al., 2003; Dias et al., 2005)
Wheat (*Triticum aestivum*)	0.19 (dimeric; WDAI-0.19; WRP24)	TMA (*K*_i_ = 0.85 nM), HSA (*K*_i_ = 0.29 nM), HPA, PPA (*K*_i_ = 57.3 nM), chicken pancreas *α*-amylase (*K*_i_ = 3.7 nM), *B. subtilis α*-amylase, and *α*-amylases from Western corn rootworm, Colorado potato beetle, sawtoothed grain beetle, red flour beetle, shield bug, and several weevil species	α-amylase cotton boll weevil, chymotrypsin or trypsin	1HSS	(O’Donnell and McGeeney, 1976; Buonocore et al., 1980, 1984; Sanchez-Monge et al., 1989; Gutierrez et al., 1990; Takase, 1994; Goff and Kull, 1995; Choudhury et al., 1996; Oda et al., 1997; Franco et al., 2000, 2005; Titarenko and Chrispeels, 2000; Oliveira-Neto et al., 2003; Oneda et al., 2004; Zoccatelli et al., 2007)
	0.28 (monomeric; WMAI-1)	TMA (*K*_i_ = 0.13 nM), HSA, and *α*-amylases from Colorado potato beetle, sawtoothed grain beetle, red flour beetle, shield bug	Chymotrypsin or trypsin		(Buonocore et al., 1980; Sanchez-Monge et al., 1989; Gutierrez et al., 1990; Choudhury et al., 1996; Payan, 2004)
	0.53	HPA, HSA, PPA (low), TMA, *Bacillus subtilis α*-amylase, and *α*-amylases from Colorado potato beetle, sawtoothed grain beetle, red flour beetle, shield bug, bean weevil, wheat weevil	α-amylases from *B. stearothermophilus, B. amyloliquefaciens, B. licheniformis, Aspergillus oryzae*, and cotton boll weevil, chymotrypsin or trypsin		(Maeda et al., 1982; Sanchez-Monge et al., 1989; Gutierrez et al., 1990; Takase, 1994; Choudhury et al., 1996; Franco et al., 2000, 2005; Oliveira-Neto et al., 2003)
	CM3	Porcine *α*-amylase (*K*_D_ = 340 nM; *K*_i_ = 600 nM), *α*-glucosidase from *Saccharomyces cerevisiae*, porcine trypsin (*K*_D_ = 36 nM; *K*_i_ = 10.4 nM)	Bovine pancreatic trypsin or HSA		(Cuccioloni et al., 2016; Thiel et al., 2020)

### 2.1 Well-Characterised CTIs

#### 2.1.1 Biochemical Properties

Several CTIs originating from different cereals have been biochemically characterized to some degree during the past decades. While their target enzyme specificity and selectivity have been tested, actual inhibition constant (*K*_i_) or binding constant (*K*_D_) values have only been reported for very few (Table 1). This is in most cases because the proteins were purified from their original source; hence, either the purity or the yield or both have been low. Recombinant protein production has enabled mutational analysis of structure/function relationships of a few CM-protein inhibitors (García-Maroto et al., 1991; Alam et al., 2001; Møller et al., 2015, 2021) and three-dimensional structures have been determined for four members including some in complex with target enzymes, namely wheat 0.19 dimeric inhibitor (Oda et al., 1997), ragi bifunctional *a*-amylase/trypsin inhibitor (RBI) (Strobl et al., 1995, 1998; Gourinath et al., 2000), corn Hageman factor/α-amylase inhibitor (CHFI) (Behnke et al., 1998), and barley limit dextrinase inhibitor (LDI) (Møller et al., 2015) (Table 1). Most characterized CTIs that inhibit hydrolases, except LDI, target *α*-1,4-glucan endo-acting *a*-amylases from GH13. By contrast, LDI from barley exclusively inhibits a debranching enzyme, limit dextrinase also belonging to GH13, that hydrolyses *α*-1,6-branch points in starch and glycogen and, in particular, *α*-limit dextrins obtained from these two branched *α*-glucans (Table 1). In addition, LDI is a special case for another reason, namely that it inhibits and hence regulates an endogenous enzyme, rather than exogenous enzymes typically from insect pests. Most CM-protein *α*-amylase inhibitors show specificity with regard to the target *α*-amylase, mainly explained by small differences in the architecture around the active site in the *α*-amylases (Rane et al., 2020). Usually, the CTIs act to a different degree against mammalian and insect digestive *a*-amylases (Table 1). Their inhibition of *α*-amylases has been shown to be influenced by the presence and type of substrate (Alam et al., 2001), and in general CTIs are not capable of completely inhibiting their target *a*-amylases (O’Donnell and McGeeney, 1976; Takase, 1994; Maskos et al., 1996; Titarenko and Chrispeels, 2000; Alam et al., 2001). Notably, RBI has been shown to bind to starch, which makes it unable to inhibit its target enzyme TMA (Alam et al., 2001). Among the characterized CTIs, LDI is the most potent. It binds its target enzyme, barley limit dextrinase, with a *K*_D_ of 42 pM mainly owing to an extremely slow off rate (Møller et al., 2015). The complex formation between LDI and barley limit dextrinase is driven by a free energy change (Δ*G*° = –57 kJ/mol) originating from equally favorable entropy and enthalpy changes (Møller et al., 2015). Wheat 0.19 AI inhibited porcine pancreatic *a*-amylase (PPA) activity by an inhibition constant (*K*_i_) of 57.3 nM, and the interaction was found to be endothermic and driven by a large increase in entropy (Oneda et al., 2004). Generally, CTIs are very stable proteins as inherent to their disulfide bonds connecting the four *a*-helix bundle (Figure 2A). The activation energy for the thermal inactivation of 0.19 AI was determined to be 87.0 kJ/mol, and *T*_50_, here the temperature causing 50% inactivation by 30 min incubation at pH 6.9, was 88.1°C (Oneda et al., 2004). RBI is stable in 8 M urea and 6 M guanidine-HCl. Notably, in 150 mM NaCl, thermal denaturation does not occur up to 90°C. However, RBI is irreversibly denatured in 5 mM NaCl if heated above 73°C. The acidic denaturation of RBI is reversible in both high and low salt conditions (Alagiri and Singh, 1993). LDI from barley is stable in the pH 2–12 range, and, at pH 6.5, its half-life is 53 and 33 min at 90 and 93 °C, respectively (Jensen et al., 2011). Furthermore, the melting temperature (*T*_m_) is 97.4 °C at pH 6.5 and the unfolding is irreversible. Notably, the inhibitor had a stabilizing effect on its target enzyme, barley limit dextrinase. The free enzyme has a *T*_m_ of 65.9°C, while the *T*_m_ of the complex is 77.4 °C (Møller et al., 2015). It is not known how LDI is released from limit dextrinase *in vivo*, but *in vitro* studies have shown that LDI can be inactivated by barley thioredoxin-catalysed disulfide reduction resulting in conformational destabilization and loss of function. Furthermore, the destabilized structure is more susceptible to protease degradation (Jensen et al., 2012).

#### 2.1.2 3D Structure of Complexes of CTIs and Amylolytic Enzymes

The four structure-determined members; LDI, RBI, wheat 0.19, and CHFI are among the best characterized CTIs (Table 1). Especially, the complex structures between LDI (Møller et al., 2015) and RBI (Strobl et al., 1998) and their respective target enzymes give unique insights into the structural basis for the function of CTIs from the different crop cereals. Besides these two published complex structures, the structure of the complex between TMA and the wheat 0.28 *α*-amylase inhibitor has been determined (Payan, 2004), but the coordinates of this structure have not been published. Prior to the determination of the structure of the complex between RBI and TMA, it was known that RBI contained two separate binding sites; one for *α*-amylase and one for protease (Maskos et al., 1996), and, moreover, that the N-terminal segment of the wheat 0.28 *α*-amylase inhibitor was crucial for its inhibitory activity (García-Maroto et al., 1991). In the RBI–TMA complex indeed, the N-terminal segment of RBI was a key element in the *α*-amylase binding site (Figure 2B), while a loop between two of the *α*-helices served as the protease-binding site (Strobl et al., 1998). Comparisons between the structures of free RBI (Protein Data Bank www.rcsb.org, PDB, entries 1B1U and 1BIP), solved both by NMR and X-ray crystallography, and RBI in complex with TMA (PDB entry 1TMQ) revealed that the N-terminal segment undergoes a conformational change upon complexation, adopting a 3_10_-helix structure, whereas it is highly flexible in the free inhibitor (Strobl et al., 1998). Notably, the unpublished complex structure between the wheat 0.28 CM-protein and TMA was reported to show the same binding features (Payan, 2004). Lastly, the protease inhibition site of RBI is a canonical substrate-like conformational region (Gly32–Thr37) situated at the opposite side of the protein. Hence, RBI can bind an *a*-amylase and a protease simultaneously. The complex structure analysis between LDI and limit dextrinase (PDB entry 4CVW) displayed an unexpected binding mode in which, unlike the other characterized CTI-*α*-amylase complexes, the N-terminal region of LDI is not interacting with the target enzyme (Figure 2B) (Møller et al., 2015). This was also confirmed by LDI N-terminal truncations showing no influence on the inhibition of limit dextrinase (Møller et al., 2015). Moreover, site-directed mutagenesis established that a hydrophobic cluster situated on the second LDI *α*-helix flanked by ionic interactions at the protein-protein interface was important for the picomolar affinity of the enzyme complex (Møller et al., 2015). Furthermore, computer-guided thorough mutational analysis of the complex revealed that LDI–limit dextrinase intermolecular contacts as well as intramolecular interactions in LDI play a role for the ultra-high affinity (Møller et al., 2021). Remarkably, the inhibitor–enzyme complexation does not rely on an interface-centered hotspot constituted by a few residues, as in the case of the other CTI *α*-amylase inhibitors. Rather LDI residues across the protein interface contributed importantly to binding, hence making the complex more robust to mutational drift in evolution (Møller et al., 2021).

### 2.2 Engineering of CTIs

Among the CTIs targeting hydrolases, only LDI and RBI have been subjected to protein engineering attempts. The complex structure between LDI and limit dextrinase provided as mentioned above a starting point for rational and computer-guided engineering of LDI showing the potential of LDI as a backbone for engineering (Møller et al., 2015, 2021). Limit dextrinase plays a key role in malting and mashing together with other endogenous amylolytic enzymes in germinated barley seeds. Thus, LDI could be considered as unwanted because it inhibits limit dextrinase and hence decreases the degradation of starch to fermentable sugars, but as mentioned above LDI also protects limit dextrinase from thermal inactivation during mashing. Besides the engineering of LDI, it has been shown that a structure-guided point mutation in barley limit dextrinase, importantly reduced the affinity for LDI without affecting the activity of the enzyme (Møller et al., 2015). Alam et al. investigated N-terminal fragments and various peptides (7–11 residues) homologous to the N-terminal sequence of RBI for their potential to inhibit PPA. The peptides all inhibited PPA catalyzed hydrolysis of the substrate *p*-nitrophenyl-α-D-maltoside more weakly as compared to RBI. Notably, however, unlike RBI, these peptides did not interact with larger substrates like starch and actually exerted a clear competitive inhibition of the hydrolysis of starch by PPA, which confirmed the potential for design of simple *a*-amylase inhibitors (Alam et al., 2001).

## 3 Applications and Impacts of CTIs in Biotechnology and Biomedicine

The individual target enzyme specificities of CTIs towards digestive *a*-amylases from insects and mammals have motivated profiling of inhibitor contents for potential cultivar selection or enrichment by using gene editing of plants to reinforce their defense against primarily insect pests (Tsvetkov and Yarullina, 2019). A recent review addresses the inhibition of different insect *a*-amylases by plant proteinaceous inhibitors including CM-proteins from wheat, rye, corn and barley (da Lage, 2018). A more practical approach consists in application of artificial diets containing either recombinantly produced inhibitors or efficient plant fractions to reduce viability of herbivorous insects (Dias et al., 2005; da Silva et al., 2013; Sagu et al., 2021). For crops, the backside of this strategy can be negative consequences of higher levels of CTIs on human health and in feed for livestock. The awareness on NCWS is important (Geisslitz et al., 2021) and durum wheat recently has been gene edited to reduce CM3 and CM16 (Camerlengo et al., 2020). Notably, CM3 treatment reduced the lifespan of *Drosophila melanogaster* fruit flies and led to bacterial overgrow in their gut, which can also be seen in humans and leading to symptoms reminiscent to NCWS (Thiel et al., 2020). On a related note, it has been reported that sourdough fermentation can degrade wheat ATIs and reduce pro-inflammatory activity (Huang et al., 2020). Quantitation using targeted multi-reaction-monitoring LC-MS/MS of ATIs in the sourdough, during proofing, and after baking, respectively, demonstrated that ATI contents were much reduced by baking (Won et al., 2021). Interestingly, recently CM-proteins are suggested to slow starch digestion rate of cooked pasta (Zou et al., 2019) in line with previous reports of wheat amylase inhibitors reducing postprandial plasma glucose concentrations (Kodama et al., 2005; Ninomiya et al., 2018) and CM-protein inhibitors 0.19, 0.28 and 0.53 being shown to effectively inhibit human pancreatic *α*-amylase (HPA) secreted into the duodenum (Choudhury et al., 1996). In brewing, the barley CM-proteins have been associated with beer-haze formation (Ye et al., 2011) and foam stability to be significantly improved by BDAI-1 (Iimure et al., 2015). Thioredoxin reduction of disulphide bonds in CTIs may influence their activity (Wong et al., 2004; Jensen et al., 2012). Barley thioredoxin h preferably reduced different disulfides in the two CM-protein inhibitors monomeric and dimeric amylase inhibitors (BMAI and BDAI), which probably has no implications for the malting and mashing, as these are directed towards exogenous *α*-amylases (Maeda et al., 2004, 2005; Jensen et al., 2012). By contrast, the inactivating reduction of disulfides in LDI (Jensen et al., 2012) may play a role during mashing, as *α*-1,6-glucosidic bond hydrolysis by limit dextrinase will be able to occur to a greater extent.

## 4 Future Perspectives

In a few cases, the structure as well as biochemical and biophysical parameters or enzyme complexation have been determined providing a basis for rational engineering of CTIs to be directed towards specific enzymes. One area of interest would be to be able to engineer CTIs to control and arrest catalysis by new enzyme targets with selected activities in cocktails of liquefying, saccharifying and debranching enzymes of family GH13, used for productions of syrups and maltooligosaccharides from starch.

Another area of emerging application is breeding and gene editing for crops to lower CTI contents to avoid allergies and still maintain resistance against pathogens. In this context, it deserves mentioning that many open questions remain to the causative role of CTIs in NCWS pathophysiology triggered by our diets (Geisslitz et al., 2021). Towards improved understanding one may use antibodies raised against reombinantly produced individual CTIs (Tundo et al., 2018). The example of sourdough baking reducing ATIs (Won et al., 2021) draws attention to examination of various cooking and other food-processing practices as a way to further develop the management of ATI contents.

## Abbreviations

AI, *α*-amylase inhibitor; ATI, α-amylase/trypsin inhibitor; BDAI, barley dimeric *α*-amylase inhibitor; BMAI, barley monomeric *α*-amylase inhibitor; CHFI, corn Hageman factor/α-amylase inhibitor; CM1 (2, 3, 16, 17), CM-protein 1 (2, 3, 16, 17) from wheat; CMa-e, CM-protein a-e from barley; CM-proteins, Chloroform:methanol soluble proteins; CTI, cereal type inhibitor; GH13, glycoside hydrolase family 13; HPA, human pancreatic *α*-amylase; HSA, human salivary *α*-amylase; LD, limit dextrinase; LDI, limit dextrinase inhibitor; NCWS, non-celiac wheat sensitivity; PDB, Protein Data Bank; PPA, porcine pancreatic *α*-amylase; RBI, ragi bifunctional *α*-amylase/trypsin inhibitor; TMA, Tenebrio molitor *α*-amylase; WCI, wheat chymotrypsin inhibitor
